# Beta and Pilot Testing of the Surviving & Thriving Healthy Lifestyle App: A Countermeasure to Firefighters’ Occupational Health Risks

**DOI:** 10.3390/toxics13030159

**Published:** 2025-02-25

**Authors:** Irene Lidoriki, Bogdan Anđelic, Fan-Yun Lan, Maria Soledad Hershey, Spyridon Georgakopoulos, Kishor Hadkhale, Eleni Speros, Mercedes Sotos-Prieto, Costas A. Christophi, Stefanos N. Kales

**Affiliations:** 1 Department of Environmental Health, Harvard T.H. Chan School of Public Health, Boston, MA 02115, USA; andjelic.bogdan92@gmail.com (B.A.); flan@hsph.harvard.edu (F.-Y.L.); kishor.hadkhale@gmail.com (K.H.); mercedes.sotos@uam.es (M.S.-P.); costas.christophi@cut.ac.cy (C.A.C.); skales@hsph.harvard.edu (S.N.K.); 2 Department of Occupational Medicine, Cambridge Health Alliance, Cambridge, MA 02145, USA; 3 Sport and Exercise Sciences Research Unit, University of Palermo, 90133 Palermo, Italy; 4 Institute of Health and Welfare Policy, National Yang Ming Chiao Tung University, Taipei 11221, Taiwan; 5 Human Flourishing Program, Institute of Quantitative Social Science, Harvard University, 12 Arrow Street, Cambridge, MA 02138, USA; mhershey@hsph.harvard.edu; 6 Hellenic Center for Excellence in Health & Wellness, Newton, MA 02459, USA; geospy.bs@gmail.com; 7 Unit of Health Sciences (Epidemiology), Faculty of Social Sciences, Tampere University, 33520 Tampere, Finland; 8 Massachusetts College of Pharmacy and Health Sciences, Boston, MA 02115, USA; efsperos01@gmail.com; 9 Department of Preventive Medicine and Public Health, School of Medicine, Universidad Autónoma de Madrid, Avda del Arzobispo Morcillo, 4, 28029 Madrid, Spain; 10 CIBERESP (CIBER of Epidemiology and Public Health), Av. Monforte de Lemos, 3-5, 28029 Madrid, Spain; 11 IMDEA-Food Institute, CEI UAM+CSIC, Ctra. de Canto Blanco 8, E, 28049 Madrid, Spain; 12 Cyprus International Institute for Environmental and Public Health, Cyprus University of Technology, Limassol 3041, Cyprus

**Keywords:** smartphone application intervention, firefighters, healthy lifestyle, occupational risk, nutrition, physical activity, sleep, resilience

## Abstract

Background: Firefighters face elevated chronic disease risks, and interventions promoting healthier lifestyles are essential for improving their well-being. This study aimed to beta test and further evaluate a healthy lifestyle app (HLS app) for firefighters. Methods: Beta usability testing was conducted with new firefighters after using the app. Pilot testing was conducted in two cohorts, (1) the Connecticut Fire Academy Class A-CCA after graduation and (2) the Connecticut Class B-CCB and Miami-Dade Fire Rescue Academy, during academy training to evaluate the potential efficacy of the HLS app in improving healthy lifestyle behaviors, mental health, and physical fitness over three months of use. Results: Beta testing (n = 93) revealed positive usability feedback, with 62% finding it useful for their health. Pilot testing after graduation (n = 28) was associated with increased push-up capacity (35.6 ± 11.7 vs. 42.9 ± 16.1, *p* = 0.006) and improved mental health scores. Pilot testing during academy training (n = 90) was associated with improvements in push-up capacity (33.8 ± 10.8 vs. 41 ± 10.6, *p* < 0.001), pull-ups (7 [4–11] vs. 10.5 [6–14], *p* < 0.001), 1.5-mile run time (11.96 ± 1.43 vs. 11.26 ± 1.1, *p* < 0.001), BMI (26.7 [24.3–29.7] vs. 25.95 [24.0–28.8], *p* < 0.001), and mental health scores. Conclusions: The app was well received and showed potential for improving firefighter health. A randomized controlled trial is needed to rigorously evaluate the effectiveness of the HLS app.

## 1. Introduction

Firefighters are a vulnerable occupational group due to their unique exposure to carcinogens, toxic substances, shift work, and other occupational hazards/stressors inherent to their profession. Additionally, more than one-third of career male firefighters are classified as obese based on their BMI, with an even higher obesity prevalence among male volunteer firefighters (45–50%) [1]. As a result of their working conditions and the high prevalence of obesity, firefighters are at increased risk of developing chronic health conditions, including cancer, cardiovascular disease (CVD), and mental health issues [2]. Obesity in firefighters is also strongly linked to chronic conditions such as metabolic syndrome (MetSyn) [3,4], CVD [5,6], and cancer [7,8,9], compounding the occupational health challenges they face. Mental health challenges, including depression, post-traumatic stress disorder (PTSD), substance use disorders, and suicide, are also prevalent in the fire service [10,11].

While firefighter training academy programs often improve recruits’ physical fitness and body composition, these gains are typically short-lived [12,13]. Within eight months of active service after graduation, many probationary firefighters experience declines in fitness and health behaviors, largely driven by sedentary work roles and unhealthy food environments within fire departments [12,13].

These physical and mental health risks underscore the urgent need for prevention strategies that can foster healthier behaviors and transform fire service culture to prioritize holistic well-being.

Workplace health promotion programs have shown effectiveness across various settings, including the fire service [14,15], while adherence to the Mediterranean lifestyle has been shown to reduce cancer mortality, the incidence of CVD, and all-cause mortality [16,17]. Similarly, physical activity interventions are associated with a lower risk of CVD, a reduction in workplace injuries, alleviation of occupational stress, and improvements in physical fitness [18,19,20]. However, firefighters often exhibit sedentary behaviors on duty [21], and structured exercise programs are rarely implemented. The demands of shift work further contribute to poor sleep quality and quantity, linked to adverse cardiometabolic and mental health outcomes [22].

Smartphone applications are increasingly popular tools for promoting healthier lifestyles and tracking habits. Several apps have been developed for first responders and public safety personnel, focusing on mental health, weight management, and behavioral health, with promising results [23,24,25,26]. However, no other app has been specifically designed to simultaneously address all the primary pillars of a healthy lifestyle—nutrition, physical activity, sleep, and resilience—while also being tailored to firefighters’ unique needs.

Over the past three years, our team has developed and refined a smartphone app intended for use as a digital healthy lifestyle (HLS) intervention for new U.S. firefighters. Initial alpha testing conducted by our study team, followed by pre-beta testing with a fire service expert panel, provided valuable feedback on the minimum viable product (MVP) to make it ‘end-user ready’. Following this work, our study aimed to beta test the newly developed app, Surviving & Thriving, which has been specifically designed for U.S. firefighters, and to evaluate its impact on healthy lifestyle scores and physical fitness among new fire recruits, exploring its potential for promoting long-term health improvements in this vulnerable occupational group.

## 2. Materials and Methods

### 2.1. Study Aims

This study has two distinct aims:

Aim 1: Beta test the HLS app with new firefighters during their academy training and probationary firefighting environments.

Aim 2: Validate the HLS app for its potential effectiveness in maintaining new firefighters’ adherence to the HLS program over 3 months and assess its associated impact on maintaining or improving HLS parameters, including lifestyle scores, physical fitness, and behavioral health (e.g., trauma and depression screens).

### 2.2. App Development

A new HLS app prototype, titled “Surviving & Thriving”, for mobile platforms (iOS/Android), was designed and developed, incorporating direct input from firefighters, insights from the existing scientific literature, and information from reliable online resources. The app aims to help firefighters implement and maintain lifestyle habits that optimize their health and functional performance [27].

The HLS app provides interactive educational content across four key lifestyle domains: nutrition, sleep, physical activity, and resilience. The app promotes balanced nutrition, regular physical activity, restorative sleep, and positive social and family connections, as well as resilience and mental health, while encouraging the avoidance of tobacco, binge drinking, and other harmful substances. The nutrition domain emphasizes balanced meal planning, healthy food choices, and sustainable eating habits, with a focus on the Mediterranean diet. The physical activity domain supports regular exercise, featuring firefighter-specific training regimens and injury prevention strategies. The sleep domain offers guidance on sleep hygiene and managing irregular shifts, while the resilience domain delivers stress management techniques and mental health support tailored to the unique challenges firefighters face.

To enhance user engagement, the app incorporates weekly motivational notifications, gamification features, a habit tracker, and team missions to promote user interaction. The final app prototype includes three self-study levels for each of the four HLS domains, with each level comprising 10 quests—120 quests in total. The app is designed to help users adopt healthy dietary habits, establish positive health behaviors, and cope with the demanding and stressful nature of their profession.

Following the initial development of the app, the first stages of testing were conducted. Internal “alpha” testing was performed by the research team (n = 10), simulating the firefighter user experience to identify potential issues and development errors in the MVP (minimum viable product) before sharing it with the target population. This was followed by “pre-beta” testing with a fire service expert panel associated with the research team (n = 10). These tests allowed the research team to refine and update the initial HLS app prototype before field testing the final prototype version.

More detailed information about the app’s development and content can be found in the previously published protocol paper [27].

### 2.3. Study Sample

New firefighters (i.e., those in fire recruit academies or who graduated from the academy less than one ago), aged 18 years or older, fluent in English, and undergoing training at our partner training sites—Connecticut Fire Academy and Miami-Dade Fire Rescue Academy—were eligible to participate in the study. Participants were enrolled after providing informed consent through internet-based consent forms on the Cambridge Health Alliance (CHA) Research Electronic Data Capture (REDCap) platform. The electronic informed consent module was used to capture actual signatures from the participants.

The study was conducted in accordance with the Declaration of Helsinki and was approved by the Institutional Review Board of the Cambridge Health Alliance (CHA-IRB-22-23-141, 16 December 2022) and the US Department of Homeland Security Compliance Assurance Program Office (22 February 2023).

### 2.4. Beta Testing

After finalizing the app prototype, beta usability testing was conducted with recent graduates from two fire academies (Connecticut and Miami-Dade Fire Rescue Academies). Participants received a link to download the app after providing consent and were instructed to explore its features, including the onboarding survey, avatar setup, educational materials, and level-up and point systems. The participants used the app for at least 2–3 weeks and then completed an 18-question online satisfaction survey adapted from a validated questionnaire to assess app functionality, as well as the strengths and weaknesses of its HLS content [28]. Additionally, at the end of the survey, participants had the opportunity to leave comments in an open-ended format. The feedback collected from the survey is used to refine the content and usability of the app and associated materials, transforming the “pre-consumer” version into an enhanced “Release Candidate” version.

### 2.5. Pilot Testing

Pilot testing was conducted at two academies, the Connecticut Fire Academy and Miami-Dade Fire Rescue Academy, to evaluate the potential efficacy of the HLS app in improving healthy lifestyle behaviors, mental health, and physical fitness over approximately three months of use. At the Connecticut Fire Academy, firefighters were recruited from two different classes. In the first class (Connecticut Class A-CCA), participants joined the study upon academy graduation, while in the second class (Connecticut Class B-CCB), participants were recruited at the beginning of their academy training and followed up with at the conclusion of the program, about three months later. At the Miami-Dade Fire Rescue Academy, participants were similarly recruited at the start of their academy training and followed up with at the end of the program, for a similar period of time. Lifestyle scores were derived from validated online questionnaires administered both before and after using the HLS app.

Changes in HLS parameters during pilot testing were assessed using repeated measures of health metrics collected pre- and post-app usage. These included data from online lifestyle and mental health questionnaires as well as fitness tests conducted before app use and again at the three-month follow-up.

### 2.6. Study Measurements

#### 2.6.1. Socio-Demographic Data, Healthy Lifestyle Behavior and Mental Health Metrics

Socio-demographic characteristics (i.e., age, sex, race, educational level) were collected using an online questionnaire administered via REDCap. The HLS score was also assessed online, consisting of seven validated components. A detailed description of the questionnaire is available elsewhere [27]. In brief, participants received 1 point for each of the following positive lifestyle behaviors: non-smoking in the past 6 months, moderate-to-high physical activity (>16 METs-h/week), high adherence to the Mediterranean diet (Mediterranean diet adherence screener score—MEDAS ≥ 9), BMI ≤ 30 kg/m^2^, limited TV watching (<2 h/day), adequate sleep (7–8 h/night), and regular napping (e.g., siesta or post-lunch nap); otherwise, they received 0 points for each item. Scores ranged from 0 (lowest) to 7 (highest), with higher scores indicating healthier lifestyle habits. The HLS score has been previously associated with a lower risk of hypertension and improved aerobic capacity among young firefighter recruits [6].

Mental health was assessed using the following validated tools: the modified Beck Depression Inventory (BDI-PC), a 6-item questionnaire with each item rated on a scale from 0 to 3 [29]; the Patient Health Questionnaire (PHQ-9), a 9-item questionnaire with each item rated on a scale from 0 to 3 [30]; and a modified version of the Posttraumatic Stress Disorder Checklist (PCL-5), a 19-item questionnaire with each item rated on a scale from 0 to 4 [31]. To mitigate the challenges of triaging “yes” responses regarding psychiatric referrals in the context of a research study (as opposed to clinical assessments), suicide/self-harm items were omitted from the BDI-PC and PCL-5.

#### 2.6.2. Physical Fitness

Physical fitness was assessed through push-up exercise capacity [19], pull-ups (maximum), and run time (1.5 miles). Push-ups were recorded as the number performed in one minute, counted continuously until the participant became exhausted, could not maintain proper form, or broke cadence. Pull-ups were counted as the maximum number completed in a single trial, with good cadence and an overhead grip. These measurements were coordinated and collected with academy training staff following the academy’s established testing protocols, except for participants who had already graduated from the academy (CCA), where the push-up capacity was remotely supervised via Google Meet by a member of the research team.

#### 2.6.3. Anthropometric and Body Composition Parameters

The measurements were performed by experienced physical trainers or other trained academy personnel, following standardized protocols. Height was measured using a clinic stadiometer (Portable Stadiometer 213, SECA, Hamburg, Germany). Body weight was assessed using a digital scale and was used to calculate the Body Mass Index (BMI) as weight (kg)/[height (m)]^2^.

### 2.7. Statistical Analysis

All data were collected using de-identified study codes. Initial data merging, cleaning, management, and basic statistical analyses were performed using SPSS 28.0 statistical software. For quantitative data (i.e., satisfaction survey data from beta testing and all data collected during the pilot phase), continuous characteristics that are normally distributed are presented as mean ± SD, and comparisons between pre- and post-app usage were made using the paired t-test. For data with skewed distributions, the results are presented as median and first and third quartiles, with comparisons made using non-parametric techniques such as the Wilcoxon signed-rank test for paired data. Dichotomous or categorical characteristics are described as frequency (%), and pre- and post-values were compared using the McNemar’s test. The primary outcome for validating the efficacy of the HLS app among new firefighters was the change in the HLS score, comparing participants’ pre- and post-scores. Individual components of the HLS score (e.g., PREDIMED score, sleep patterns, and physical activity) were also analyzed.

## 3. Results

### 3.1. Beta-Testing

**Figure 1:**
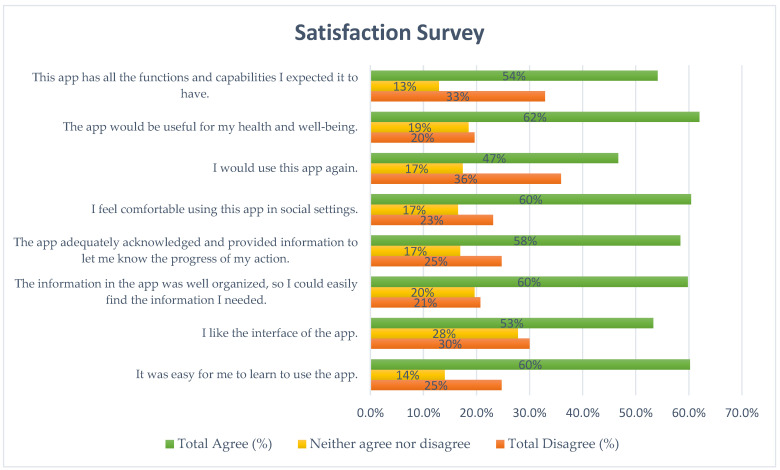
Satisfaction survey results across main question categories for the beta-testing group.

Ninety-three participants from two different academies (Connecticut and Miami-Dade Fire Rescue Academy) completed the satisfaction survey (beta-testing group). Responses were categorized into three groups: Total Agree, which included answers 5 (somewhat agree), 6 (agree), and 7 (strongly agree); Neither Agree nor Disagree, which included answer 4; and Total Disagree, which included answers 1 (strongly disagree), 2 (disagree), and 3 (somewhat disagree). Responses not answered or marked as “NA” were treated as missing data. According to the results (Figure 1), 60% of participants reported that it was easy for them to learn to use the app, 53% liked the app’s interface, and 60% found the information well-organized and easy to navigate. Additionally, 60% of respondents felt comfortable using the app in social settings, and 62% agreed that the app would be useful for their health and well-being. However, 36% of participants would not use the app again, 44% did not find it helpful for managing their health effectively, and 38% were not satisfied with the app. The full questionnaire responses, including NA responses, are provided in Supplementary Table S1.

### 3.2. Pilot-Testing

Baseline sociodemographic data for both academies are presented in Table 1. Results are presented separately for Connecticut Class A (CCA) (Table 2) after-graduation use, and for Connecticut Class B (CCB) and the Miami-Dade Fire Rescue Academy combined (Table 3) during-academy use.

#### 3.2.1. App-Use After Graduation

A total of 63 participants from CCA were initially included in the study, with 28 participants completing the follow-up assessment after graduation, corresponding to three months of app use. Among the participants who completed the follow-up, the mean age was 29.3 ± 5.1 years, and 92.9% were male.

Following app use, there was a statistically significant increase in push-up capacity (35.6 ± 11.7 vs. 42.9 ± 16.1, *p* = 0.006) and a statistically significant decrease (improvement) in PCL-5 scores (24.5 [21.0–31.0] vs. 21.5 [20.0–25.5], *p* = 0.007) and PHQ-9 scores (10.00 [9.25–13.00] vs. 9.00 [8.00–10.75], *p* < 0.001) (Table 2, Supplementary Figure S1). In contrast, BMI significantly increased (28.6 ± 4.0 vs. 29.4 ± 4.4 kg/m^2^, *p* = 0.006).

#### 3.2.2. App-Use During the Academy Graduation

Initially, 96 included in the study, with 90 participants completing the follow-up assessment (CCB: 60, Miami-Dade Fire Rescue Academy: 30). Among those who completed the follow-up, the mean age was 27.4 ± 6.1 years, with 93.3% being male (Table 1).

Post app use (Table 3, Supplementary Figure S2), there was a statistically significant improvement in physical performance metrics, including an increase in push-up capacity (33.8 ± 10.8 vs. 41 ± 10.6, *p* < 0.001), pull-up capacity (7 [4–11] vs. 10.5 [6–14], *p* < 0.001), and a reduction in 1.5-mile run time (11.96 ± 1.43 vs. 11.26 ± 1.1, *p* < 0.001). BMI significantly decreased (26.7 [24.3–29.7] vs. 25.95 [24.0–28.8], *p* < 0.001). Mental health scores improved significantly, with reductions in both BDI-PC (0 [0–1.75] vs. 0 [0–1], *p* = 0.007) and PCL-5 (5 [2–12.75] vs. 3 [1–9], *p* < 0.001). Additional findings included an increase in physical activity levels, with the proportion of participants engaging in ≥16 MET-h/week rising from 56.7% to 70% (*p* = 0.037). Napping behavior changed significantly, with all participants reporting napping at follow-up (*p* = 0.002).

## 4. Discussion

A new HLS smartphone application, titled “Surviving & Thriving”, specifically tailored for new firefighters, was developed by integrating feedback from firefighters, findings from current scientific research, and data from reliable online sources. This study beta tested the HLS app and evaluated its impact on healthy lifestyle scores and physical fitness, as well as its potential to promote long-term health improvements within this occupational group. Our findings indicate that the app was well received by the fire recruits, and was associated with significant improvements or stability in multiple health metrics with several months of App use.

Firefighters face elevated chronic disease risks, with research consistently indicating a high prevalence of obesity and other cardiometabolic issues, with adverse effects on health, performance, safety, and associated costs [21]. Notably, these include a high proportion (50%) of on-duty deaths attributed to cardiovascular disease (CVD) [8,32,33] and an estimated 10–15% increase in the lifetime risk of developing or dying from a serious cancer diagnosis [9]. Although occupational factors contribute to these conditions, lifestyle-related diseases account for two-thirds of lifetime mortality among U.S. firefighters [10]. Cardiac enlargement is a significant risk factor for on-duty, sudden cardiac death, and lifestyle-related conditions such as hypertension, obesity, and obstructive sleep apnea contribute to increased heart weight/cardiomegaly [34,35]. Moreover, while firefighters are known to be exposed to several carcinogens [36,37,38,39], it is well documented that cancer risk can be mitigated through a healthy diet, regular physical activity, maintaining a healthy weight, and avoiding tobacco and excessive alcohol [40,41,42,43]. Additionally, healthy lifestyle behaviors can reduce the risks and improve the symptoms of depression, post-traumatic stress, and other mental health issues, which also pose significant concerns for the fire service [12,13,21].

For a lifestyle intervention to be effective, it must be acceptable to the target population. The Surviving and Thriving App was created based on several years of work with fire service members and leaders. Initial qualitative research highlighted the importance of fostering a cohesive culture among fire academy staff and recruits to support long-term lifestyle changes [44]. A healthy lifestyle app directed at fire academy recruits and recent graduates was identified as a potentially cost-effective approach. After successfully proposing the HLS app and developing a minimum viable product, we conducted internal testing as well as pre-beta testing with fire service leaders in order to create the prototype version of the app [27].

In this study, our first aim was conducting rigorous beta testing of the prototype app with an 18-question satisfaction survey focused on user experience, satisfaction, the app’s functionality, and perceived health benefits. The satisfaction survey demonstrated that the HLS app was generally well received by users, with several key strengths identified. The majority of participants agreed that the app was easy to use (52%) and easy to learn how to use (60%) and had an appealing design (53%). Users found the app’s content information to be well organized (59.8%) and noted that it effectively acknowledged their actions and provided progress updates (58%). Importantly, the app was recognized for its potential health benefits, with 62% of the users considering it useful for their health and well-being. These findings highlight the app’s strengths in terms of usability, functionality, and health-related benefits, while also suggesting areas for potential refinement to further enhance user satisfaction.

It should also be noted that during beta testing, some participants experienced technical issues with the app. Participants’ open-ended comments indicated that internet connectivity issues affected their access to the app and, consequently, their overall satisfaction. While participants emphasized the need to resolve these connectivity issues, they also expressed high satisfaction with the app’s content and underlying rationale. This discrepancy is reflected in the differing satisfaction rates between participants who experienced connectivity issues within the academy and those who did not. Moving forward, we plan to collaborate with the developers to address these challenges, including exploring the possibility of enabling offline functionality to improve user experience.

The second aim of this study was to explore the HLS app’s potential ability to maintain or improve health metrics among new firefighters both during academy training and post-graduation. The results during academy training (CCB and Miami-Dade Fire Rescue Academy cohorts) demonstrated significant improvements in physical fitness metrics associated with the app intervention. Push-up capacity significantly increased (+7; *p* < 0.001) and 1.5-mile run times improved overall by 0.70 min (*p* < 0.001). Significant gains were also observed in pull-up performance. BMI decreased significantly post-intervention, with a reduction in the proportion of participants classified as obese (BMI ≥ 30) from 24.4% to 17.8%. Additionally, physical activity levels improved, with a higher proportion of participants achieving ≥16 MET-h/week (*p* = 0.037), as well as improved napping habits. Mental health metrics also showed significant improvements, including reductions in BDI-PC (*p* = 0.007) and PCL-5 scores (*p* < 0.001), suggesting benefits for depressive and PTSD symptoms. In contrast, a previous study of ninety-two New England fire recruits without app use found no significant improvements in BMI or mental health scores (BDI-PC, PCL-5, PHQ-9, all *p* ≥ 0.05) at academy graduation [12].

The positive associations with app use extended beyond academy graduation. However, it is important to note that post-app data were available for only 44.4% of the CCA cohort, which may influence the results for this group. Findings from the CCA cohort highlighted notable changes in physical and mental health metrics three months post-graduation following the use of the HLS app. The mental health scores demonstrated significant improvements, with reductions observed in PCL-5 and PHQ-9 scores, suggesting positive effects on PTSD and depressive symptoms. However, mean BMI increased slightly (+0.85 kg/m^2^; *p* = 0.006), reflecting potential lifestyle changes post-training, consistent with the previous New England cohort study without App use. Push-up capacity significantly improved overall (+7.4; *p* = 0.006), in contrast to the previous New England cohort study, where the average push-up capacity significantly decreased after a minimum of six months post-graduation [12].

We found no improvement in adherence to the Mediterranean diet (MEDAS) in the CCB and Miami-Dade Fire Rescue Academy cohorts as it slightly declined during training, whereas the scores remained stable in the CCA cohort post-graduation. Lastly, the overall HLS scores demonstrated a small but significant improvement (*p* = 0.03) during academy training, suggesting that the app selectively influenced positive changes in healthy lifestyle behaviors for some participants. Post-graduation HLS scores remained stable in the CCA cohort, which is considered a positive outcome given the previously observed tendency for lifestyle scores to decline after graduation [12].

Our current results highlight the app’s potential to enhance physical performance and mental health outcomes. However, improvements in physical performance and mental health outcomes cannot be solely attributed to the app, as the rigorous training curriculum inherently enhances recruits’ fitness. Our pilot study could not isolate the app’s specific impact due to potential confounding factors. Nonetheless, participants using the app post-academy exhibited notable improvements, indicating that the HLS app may have helped maintain and enhance fitness outcomes over time, despite the usual post-graduation decline [3,4].

Although the study did not specifically investigate the applicability of the HLS app to firefighters from different cultural backgrounds, the participant demographics reflect a diverse sample, including Caucasian, African American, Asian, and Hispanic individuals. This suggests that the app may be applicable to firefighters from various racial and ethnic backgrounds. Nonetheless, further research would be needed to assess its effectiveness and cultural adaptability in a more diverse cohort.

Various technological interventions, including mobile apps, wearables, and digital tools, have been developed to enhance the physical and mental well-being of first responders with somewhat promising results [24,25,26,45]. However, these interventions often focus on single aspects of health. To the best of our knowledge, no existing app comprehensively integrates all four key lifestyle parameters—nutrition, physical activity, resilience, and sleep—specifically for firefighters. This underscores the innovative nature of our app, which aims to holistically support the unique health needs of this critical population.

Our study does have several limitations that should be acknowledged. Besides our limited ability to examine the app’s specific effects on health outcomes, technical challenges during beta testing, including internet connectivity issues, hindered consistent app access, particularly in areas with limited network coverage, highlighting the need for offline functionality to improve usability. Also, there were significant variations in follow-up rates, 93.8% vs. 44.4% for during and post-academy use, respectively. The lower follow-up rates post-graduation could be attributed to the challenges of maintaining engagement in less structured, real-world settings, where participants face competing personal and professional demands. This pattern is consistent with the follow-up rates observed in the previous New England cohort study [12]. Additionally, participants engaged following graduation may lack the same level of motivation or institutional support available to those given access to the app during academy training. These findings suggest that providing app access during the structured environment of academy training may foster higher engagement and compliance.

This preliminary study provides valuable insights into the feasibility, user satisfaction and potential impact of a tailored, digital lifestyle intervention for firefighters and underscores the importance of addressing technical and contextual factors in future iterations of the app. Our findings also provide a strong rationale for conducting a cluster-randomized trial across consecutive classes from various fire academies to rigorously evaluate the HLS app’s effectiveness (compared to usual care) for influencing and sustaining academy training-derived health benefits during firefighters’ first year on the job following academy or other fire training.

## Figures and Tables

**Table 1:** Baseline sociodemographic data of study participants.

Variable	Baseline	
	3-month Follow-up participants, CCA, n = 28	3-month Follow-up participants, n = 90 (CCB n = 60 and Miami-Dade Fire Rescue Academy n = 30)
Age (years)	29.3 ± 5.1	27.4 ± 6.1
Sex Male Female	26 (92.9%) 2 (7.1%)	84 (93.3%) 6 (6.7)
Race Caucasian African American Asian	26 (92.9%) 2 (7.1%) 0 (0)	78 (86.7%) 7 (7.8%) 5 (5.6%)
Hispanic No Yes	25 (89.3%) 3 (10.7%)	61 (67.8%) 29 (32.2%)
Educational level High School or Equivalent Vocational/Technical College/Associate Degree Bachelor’s Degree Master’s Degree or higher	4 (14.3%) 2 (7.1%) 7 (25%) 12 (42.9%) 3 (10.7%)	26 (28.9%) 3 (5.2%) 23 (25.6%) 18 (31.0%) 3 (5.2%)

**Table 2:** Pre-and post-app data for the CCA cohort.

Variable	Pre-App (Graduation)	Post-App (3 Months Post-Graduation)		
n = 28	Mean ± SD Median (Q1–Q3)	Mean ± SD Median (Q1–Q3)	Mean Difference	*p*-Value
Push-up capacity Total (n = 26)	35.6 ± 11.7	42.9 ± 16.1	+7.4	0.006
HLS score (n = 25)	5.00 (4.00–6.00) *	5.00 (4.00–5.00) *	+0.35	0.626
BMI (kg/m^2^)	28.6 ± 4.0	29.4 ± 4.4	+0.85	0.006
BMI (kg/m^2^) ≥30 <30	7 (28%) 18 (72%)	8 (32%) 17 (68%)		1.000
MEDAS	5.5 (4–7) *	6 (4–8) *	+0.5	0.285
Smoking No (never/Former > 6 months) Yes	27 (96.4%) 1 (3.6%)	28 (100%) 0 (0)		1.000
TV watching <2 h/d ≥2 h/d	13 (46.4%) 15 (53.6%)	11 (39.3%) 17 (60.7%)		0.754
Sleep 7–8 h/d Less/more than 7–8 h/d	19 (67.9%) 9 (32.1%)	15 (53.6%) 13 (46.4%)		0.289
Nap Yes No	28 (100%) 0 (0)	28 (100%) 0 (0)		-
Physical activity ≥16 MET-h/wk <16 MET-h/wk	22 (78.6%) 6 (21.4%)	18 (64.3%) 10 (35.7%)		0.289
Mental health scores BDI-PC PCL-5 PHQ-9	7.00 (6.00–7.75) * 24.50 (21.00–31.00) * 10.00 (9.25–13.00) *	6.00 (6.00–7.00) * 21.50 (20.00–25.50) * 9.00 (8.00–10.75) *	−0.59 −2.42 −0.95	0.057 0.007 <0.001

Mean ± SD or Median (Q1–Q3) were used for paired *t*-test or paired Wilcoxon rank test, respectively. * Median (Q1–Q3). HLS, Healthy Lifestyle Score; BMI, Body Mass Index; MEDAS, Mediterranean diet adherence screener score; BDI-PC, Beck’s depression inventory for primary care; PCL-5, Posttraumatic Stress Disorder Checklist for DSM-5; PHQ-9, Patient Health Questionnaire.

**Table 3:** Pre-and post-app data for the CCB and Miami-Dade Fire Rescue Academy cohorts.

Variables	Pre-App	Post-App		
n = 90	Mean ± SD Median (Q1–Q3)	Mean ± SD Median (Q1–Q3)	Mean Difference	*p*-Value
Push-up capacity (n = 89)	33.82 ± 10.8	41 ± 10.6	+7.1	<0.001
1.5 mile run time (min) (n = 89)	11.96 ± 1.43	11.26 ± 1.1	−0.70	<0.001
Pull-up (n = 88)	7 (4–11) *	10.5 (6–14) *	+2.5	<0.001
HLS score (n = 88)	5.0 (4.0–5.0) *	5.0 (4.0–6.0) *	+0.30	0.03
BMI (kg/m^2^)	26.7 (24.3–29.7) *	25.95 (24.0–28.8) *	−0.69	<0.001
BMI (kg/m^2^) ≥30 <30	22 (24.4%) 68 (75.6%)	16 (17.8%) 74 (82.2%)		0.07
MEDAS (n = 88)	6.67 ± 2.53	6.06 ± 2.12	−0.61	0.001
Smoking No (Never/Former > 6 months) Yes	90 (100%) 0 (0%)	90 (100%) 0 (0%)		1.00
TV watching <2 h/d ≥2 h/d	60 (66.7%) 30 (33.3%)	69 (76.7%) 20 (23.3%)		0.109
Sleep 7–8 h/d Less/more than 7–8 h/d	49 (54.4%) 41 (45.6%)	50 (55.6%) 40 (40.4%)		1.00
Nap Yes No	79 (87.8%) 11 (12.2%)	90 (100%) 0 (0)		0.002
Physical activity ≥16 MET-h/wk <16 MET-h/wk	51 (56.7%) 39 (43.3%)	63 (70%) 27 (30%)		0.037
Mental health scores BDI-PC PCL-5 PHQ-9	0 (0–1.75) * 5.00 (2.00–12.75) * 2.00 (0–4.00) *	0 (0–1) * 3.00 (1.00–9.00) * 1 (0–3.00) *	−2.32 −0.36 −0.54	0.007 <0.001 0.139

Mean ± SD or Median (Q1–Q3) were used for paired *t*-test or paired Wilcoxon rank test, respectively. * Median (Q1–Q3). HLS, Healthy Lifestyle Score; BMI, Body Mass Index; MEDAS, Mediterranean diet adherence screener score; BDI-PC, Beck’s depression inventory for primary care; PCL-5, Posttraumatic Stress Disorder Checklist for DSM-5; PHQ-9, Patient Health Questionnaire.

## Data Availability

The original contributions presented in this study are included in the article/Supplementary Material. Further inquiries can be directed to the corresponding author(s).

## Supplementary Materials

The following supporting information can be downloaded at: https://www.mdpi.com/article/10.3390/toxics13030159/s1, Figure S1: Boxplots illustrating the mean difference pre- and post-use of the HLS app for statistically significant variables in the CCA cohort; Figure S2: Boxplots illustrating the mean difference pre- and post-use of the HLS app for statistically significant variables in the CCB and Miami-Dade Fire Rescue Academy cohorts; Table S1: Satisfaction survey.
