# Supplementary material for: Beta and Pilot Testing of the Surviving & Thriving Healthy Lifestyle App: A Countermeasure to Firefighters’ Occupational Health Risks
Source: Toxics. 2025 Feb 25;13(3):159. doi: 10.3390/toxics13030159 (PMC11945949; doi:10.3390/toxics13030159)
Supplement: Supplementary file 1 [file toxics-13-00159-s001.zip › toxics-3424063-supplementary.pdf]

*Supplementary Table S1. Satisfaction Survey*

| Question (1-18) (n=93) | Total Agree (n, %)[1] | Neither agree nor disagree (n, %)[2] | Total Disagree (n, %)[3] | NA |
|---|---|---|---|---|
| 1. The app was easy to use. | 48 (52%) | 13 (14%) | 31 (33%) | 1 (1%) |
| 2. It was easy for me to learn to use the app. | 56 (60%) | 13 (14%) | 23 (25%) | 1 (1%) |
| 3. The navigation was consistent when moving between screens. | 48 (52%) | 15 (16%) | 30 (32%) | 0 (0) |
| 4. The interface of the app allowed me to use all the functions (such as entering information, responding to reminders, viewing information) offered by the app. | 43 (46%) | 15 (16%) | 32 (35%) | 3 (3%) |
| 5. Whenever I made a mistake using the app, I could recover easily and quickly. | 39 (42%) | 18 (19%) | 30 (32%) | 6 (7%) |
| 6. I like the interface of the app. | 48 (52%) | 15 (16%) | 27 (29%) | 3 (3%) |
| 7. The information in the app was well organized, so I could easily find the information I needed. | 55 (59%) | 18 (20%) | 19 (20%) | 1 (1%) |
| 8. The app adequately acknowledged and provided information to let me know the progress of my action. | 52 (56%) | 15 (16%) | 22 (24%) | 4 (4%) |
| 9. I feel comfortable using this app in social settings. | 55 (59%) | 15 (16%) | 21 (23%) | 2 (2%) |
| 10. The amount of time involved in using this app has been fitting for me. | 42 (45%) | 14 (15%) | 30 (32%) | 7 (8%) |
| 11. I would use this app again. | 43 (46%) | 16 (17%) | 33 (36%) | 1 (1%) |
| 12. Overall, I am satisfied with this app. | 36 (39%) | 16 (17%) | 35 (38%) | 6 (6%) |
| 13. The app would be useful for my health and well-being. | 57 (61%) | 17 (18%) | 18 (20%) | 1 (1%) |

| | | | | |
|---|---|---|---|---|
| 14. The app improved my access to a healthy lifestyle. | 40 (43%) | 14 (15%) | 32 (34%) | 7 (8%) |
| 15. The app helped me manage my health effectively | 37 (40%) | 12 (13%) | 38 (41%) | 6 (6%) |
| 16. This app has all the functions and capabilities I expected it to have. | 46 (49%) | 11 (12%) | 28 (30%) | 8 (9%) |
| 17. I could use the app even when the Internet connection was poor or not available. | 30 (32%) | 10 (11%) | 41 (44%) | 12 (13%) |
| 18. This HLS app provides an acceptable way to receive a healthy lifestyle, such as accessing educational materials, tracking my own activities, and performing self-assessment. | 43 (46%) | 18 (20%) | 27 (29%) | 5 (5%) |

*NA: Not Applicable*

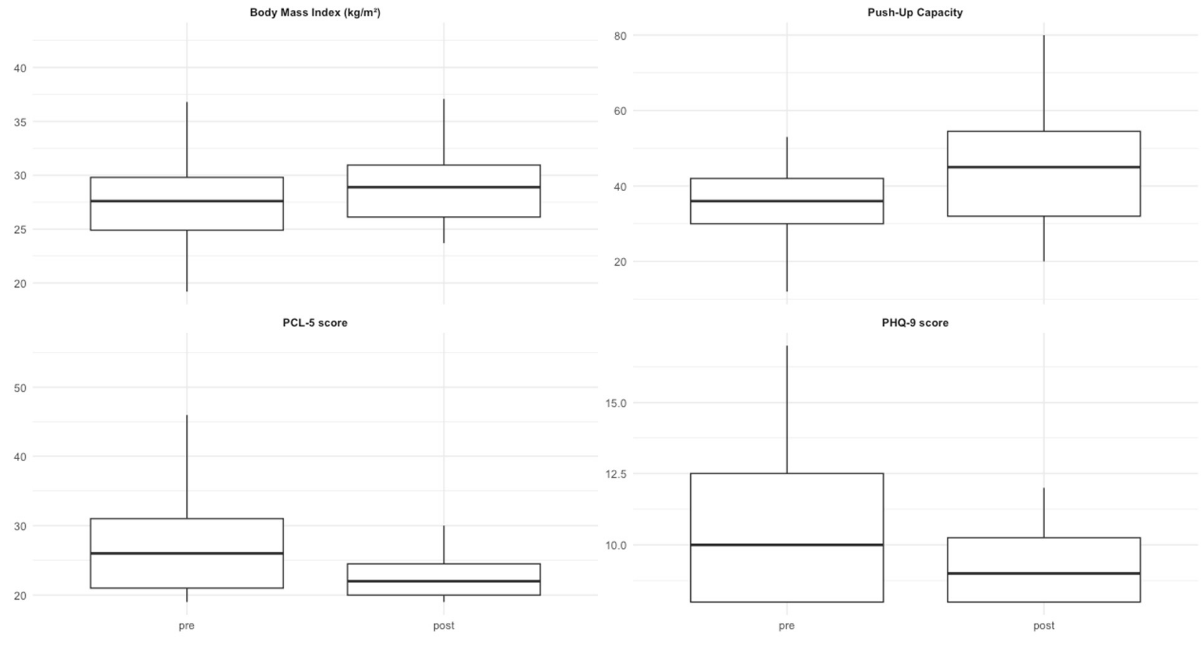


*Supplementary Figure S1. Boxplots illustrating the mean difference pre- and post-use of the HLS app for statistically significant variables in the CCA cohort.*

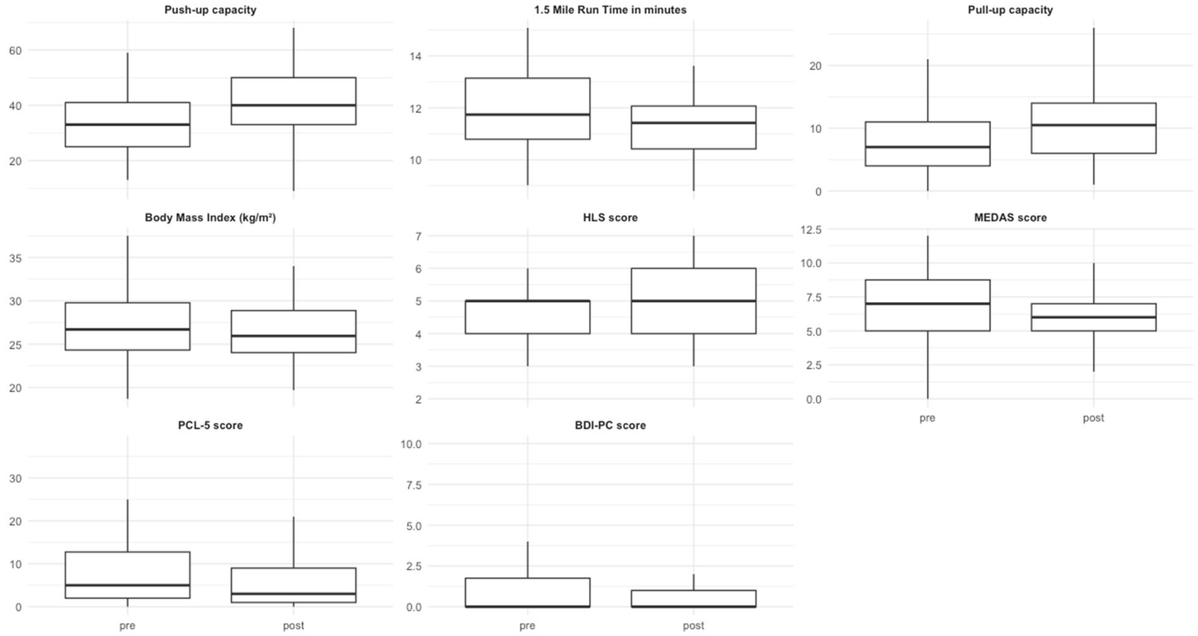


*Supplementary Figure S2. Boxplots illustrating the mean difference pre- and post-use of the HLS app for statistically significant variables in the CCB and Miami-Dade Fire Rescue Academy cohorts*
